# Systemic Sclerosis: Highlighting Respiratory Complications and Significance of Early Screening

**DOI:** 10.7759/cureus.17291

**Published:** 2021-08-18

**Authors:** Naqvi Syed Gaggatur, Aliya H Sange, Natasha Srinivas, Mubashira K Sarnaik, Srimy Modi, Yasaswi Pisipati, Sarayoo Vaidya, Ibrahim Sange

**Affiliations:** 1 Internal Medicine, M. S. Ramaiah Medical College, Bangalore, IND; 2 Research, K. J. Somaiya Medical College, Mumbai, IND; 3 Research, B. G. S. Global Institute of Medical Sciences, Bangalore, IND; 4 Research, California Institute of Behavioral Neurosciences & Psychology, Fairfield, USA; 5 Medicine, K. J. Somaiya Medical College, Mumbai, IND

**Keywords:** systemic sclerosis, pulmonary artery hypertension, interstitial lung disease, respiratory complications, screening and diagnosis, prognosis, mortality, pulmonary artery vasoconstriction, vascular remodeling, thrombosis

## Abstract

Systemic sclerosis (SSc) is an autoimmune disease that leads the patient to have a diverse clinical presentation encompassing several systems and a worse prognosis, mainly when complications arise. Most SSc-related deaths are caused by pulmonary hypertension (PH) and interstitial lung disease (ILD). This article focuses on pulmonary artery hypertension (PAH) and ILD as pulmonary consequences of SSc. We examined the grave effects regarding SSc's respiratory complications, which are concealed by the disease's clinical heterogeneity. In this article, we briefly reviewed the discussion of clinical features and management and the mortality associated with the sequelae. We further addressed the benefits and significance of screening for the disease and associated respiratory complications in SSc patients in this study.

## Introduction and background

Systemic sclerosis is an autoimmune disease that continues to challenge the medical fraternity due to its disparate nature [1]. A disease hallmarked by eventual fibrosis of the skin and the organ systems preceded pathologically always by the sequela of endothelial dysfunction resulting in disruption of the small vessel vasculature, immunological, and fibroblast dysfunction [1].

SSc is an uncommon autoimmune disease with a high case-specific mortality rate and an annual incidence of roughly 20 cases per million [2,3]. According to several studies, the incidence is higher and the prognosis is worse in African Americans as compared to Caucasians with a higher preponderance in females [2-5]. Other risk factors include Raynaud's phenomenon, steroid hormone imbalance, and selected chemical and thermal injuries [6]. The disease is characterized by a specific or functional antibody production as a response to a complex innate and adaptive immunity and involves complex pathogenesis comprising of vascular and immune dysregulation, which is inadequately comprehended [7]. The diffuse evolution of SSc involves progenitor-circulating cells such as monocytes and fibrocytes and the participation of growth factors and cytokines [7].

SSc has a diverse clinical presentation involving the skin and organs such as the lungs, heart, gastrointestinal tract, and kidneys [8]. Based on the amount of skin fibrosis involved, SSc is classified into limited cutaneous systemic sclerosis (lcSSc) or diffuse cutaneous systemic sclerosis (dsSSc) [8]. lcSSc presents with fibrosis of the skin involving the peripheral parts of the body, face, and limbs (distal to the knees and elbows), whereas dsSSc presents with fibrosis of the trunk and proximal parts of the limbs [9]. The workup of the disease is specific to the system or phenotype predominantly involved. The frequently detected antibodies are antinuclear antibodies (ANA) and autoantibodies against topoisomerase I (Topo I, also known as ATA or Scl-70) [9]. The management of this disorder is hindered by its miscellaneous presentation and an unfavorable prognostic course [10].

Most deaths are due to the complications caused by PH and interstitial lung disease (ILD) as the involvement impacts all elements of the respiratory system, including the parenchyma, muscle, and vasculature, and can occur in both categories of the disease [11-13]. SSc-associated ILD is a dreaded complication that has risen to the top of the SSc-related causes of mortality, accounting for 35% of all disease-related deaths [13]. Similarly, PAH, the other major complication of SSc, affects about 10% of the population [14]. Despite recent breakthroughs in treatment, the survival rates for the above complications remain dismal, especially for SSc-PAH patients with a median survival period of only three years [13,14]. Given such poor long-term outcomes, it is logical and imperative to screen early disease manifestations in the aforementioned illnesses. Albeit, according to new research, early screening programs in SSc and ILD can detect patients with milder forms of the disease, allowing for early therapeutic intervention and improved survival [13-15].

This review article aims to:

1. Emphasize the growing concern about SSc's life-threatening complications, which are masked by the disease's clinical heterogeneity.

2. Evaluate the importance and highlight the benefits of early screening of ILD and PAH in SSc.

## Review

The fundamental concern of researchers looking into mortality associated with systemic sclerosis (SSc) is progressive pulmonary deterioration since pulmonary complications have replaced scleroderma renal crisis as the primary cause of SSc-related death [16]. SSc can cause anything from minor parenchymal fibrosis to severe progressive ILD or isolated PH in the lungs, which can escalate to PH and aggravate the problem [17]. ILD and pulmonary arterial hypertension (PAH) are the leading causes of death in patients with systemic autoimmune disorders like SSc [18].

Systemic sclerosis-associated pulmonary arterial hypertension (SSc-PAH)

The World Health Organization (WHO) divided PH into groups based on the disease's pathogenesis, with group 1 being referred to as PAH [19]. PAH is defined as a mean pulmonary arterial pressure (mPAP) ≥ 25 mm Hg at rest, a mean pulmonary-capillary wedge pressure or left ventricular end-diastolic pressure ≤ 15 mm Hg, and a pulmonary vascular resistance (PVR) ≥ 3 Wood units (WU) [19]. PAH is a dreaded complication as it has high mortality and morbidity rates, especially as a complication in SSc, affecting 8%-15% of the SSc patients [20,21].

Manifestations of PAH are due to the combined actions of pulmonary artery vasoconstriction, vascular remodeling, and thrombosis that cause a prolonged increase in PVR and pulmonary arterial pressure (PAP) [22]. The pathophysiological mechanism is that all the sheaths of the vessel wall and all the cell types that make up the vessel wall are involved in vascular remodeling (endothelial cells, smooth muscle cells, fibroblasts, inflammatory cells, and platelets) [22]. Growth, apoptosis, mild inflammation, vascular ions, and fibrosis are dynamic processes that might affect the level of remodeling [22]. The deficiency of potassium channel expression and endothelial dysfunction has been linked to excessive vasoconstriction [22]. These effects further culminate in chronic insufficiency of vasodilators, such as nitrogen monoxide and prostacyclin, and overproduction of vasoconstrictors, such as endothelin-1 [22]. The overproduction of vasoconstrictors leads to chronic vasoconstriction, eventually resulting in smooth muscle hypertrophy and endothelial proliferation, culminating in a narrow vascular lumen with increased pressure [23].

**Figure 1:**
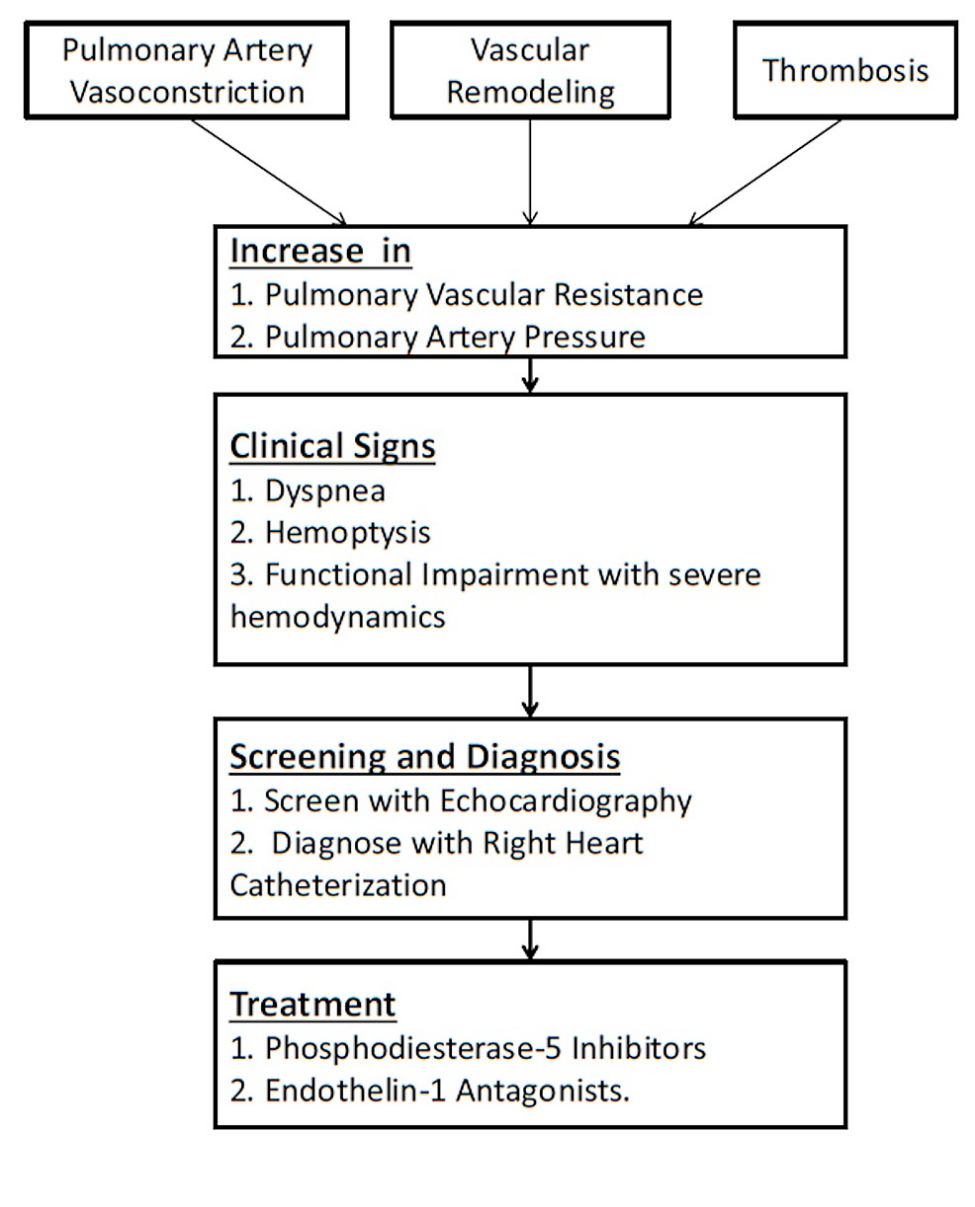
Summary of pathogenesis, investigation, and management of pulmonary artery hypertension (PAH) in systemic sclerosis (SSc), SSc-PAH

At the time of diagnosis, most patients present with severe signs and symptoms, including dyspnea, hemoptysis, or syncope, and considerable functional impairment often associated with severe hemodynamics (Figure 1) [24]. Right heart catheterization is used as a standard for diagnosing PH, and echocardiography is the screening tool where the tricuspid regurgitation velocity (TRV) and right atrial pressure are used as determinants [25]. After undergoing the initial screening, patients who tested positive for the disease underwent a right heart catheterization to confirm the diagnosis [26].

Experts continue to believe that phosphodiesterase 5 (PDE5) inhibitors, endothelin receptor antagonists with PDE5 inhibitors, and prostanoids are the best therapy for mild cases of PAH [27]. For severe cases, prostanoids are the first line of treatment [27]. Drugs such as sildenafil, a phosphodiesterase-5 (PDE-5) inhibitor, has demonstrated an increased exercise tolerance in patients with PH when used in the treatment of SSc-PAH [28]. Similar improvements in efficacy were seen with bosentan, an endothelin-1 receptor antagonist [29]. The addition of epoprostenol, a potent, short-acting vasodilator, tends to improve the six-minute walk distance and lower PVR in PH patients [30].

PAH is a feared complication, affecting around 8%-15% of SSc patients [20,21]. Any delay in diagnosing PAH in SSc patients will lead to poor prognosis; hence, studies recommending early detection and immediate treatment are critical for successful management with better prognostic outcomes [31] (Table 1).

**Table 1:** Summary of included studies linking PAH and SSc DLCO, Diffusing capacity of the lungs for carbon monoxide; ILD, interstitial lung disease; NYHA FC, New York Heart Association Functional Classification; PAH, pulmonary arterial hypertension; SSc, systemic sclerosis; SSc-PAH, systemic sclerosis-associated pulmonary arterial hypertension.

References	Design	Cases of SSc-PAH	Study parameters	Conclusion
Morrisroe et al. (2016) [34]	Cohort study	132 (Total cases of SSc was 1579.)	Incidence, prevalence, and risk factors	PAH is the leading cause of SSc-related deaths. SSc-PAH is connected to mild ILD in both categories. Early diagnosis and treatment of PAH in SSc patients might improve their prognosis.
Chung et al. (2014) [31]	Prospective multicenter cohort	131	Survival rates and mortality predictors	With the routine screening of PAH in SSc-PAH patients, the survival rates were better than those reported in similar cohorts. The patients with severely reduced DLCO and NYHA FC IV status at the time of PAH diagnosis had a dismal prognosis.
Launay et al. (2013) [32]	Prospective multicenter cohort	85	Survival and Prognostic Factors	Even with modern medical advancements, incident SSc-associated PAH continues to be a terrible disease. In this group of patients, age, male gender, and cardiac index were the most important prognostic markers. Early diagnosis of PAH in SSc patients should be a top concern.

A cohort study was performed in 2016 on a group of adults in Australia, which aimed to determine the incidence, prevalence, and risk factors for the development of PAH in SSc. In the study, out of 1579 cases of SSc, 132 cases were diagnosed to have SSc-PAH using right heart catheterization. The study concluded that PAH is the leading cause of SSc-related death, and SSc-PAH is connected to mild ILD in both subtypes. The study also emphasized that early diagnosis and treatment of PAH in SSc patients may improve their prognosis (Table 1) [31].

In another prospective multicentric cohort study done in the United States, 131 SSc-PAH patients were diagnosed using right heart catheterization as a criterion and studied. The study indicated that the survival rates were better when compared to other similar cohorts and highlighted the need for early screening and diagnosis of PAH in SSc as the prognosis is dismal on delayed diagnosis (Table 1) [32].

Similarly, another study was conducted by Launay et al. in 2013 in France, where 85 patients were diagnosed with SSc-PAH and examined for survival and prognostic factors. The study concluded that the presence of PAH in SSc patients is associated with increased mortality rates, especially in the elderly and male gender (Table 1) [33]. Hence it is critical to use early screening procedures to detect PAH in patients with SSc through routine systematic screening of asymptomatic patients. It will lead to earlier diagnosis, improved survival rates, and prognosis [32,33].

The existing annual screening protocols that are recommended utilize algorithms that comprise a transthoracic echocardiogram (TTE) and pulmonary function tests (PFTs) [34]. Screening by DETECT algorithm, pulmonary function testing (forced vital capacity/diffusing capacity of the lung for carbon monoxide ratio), and N-terminal-pro-brain natriuretic peptide levels are also used as alternatives [35]. Other screening modalities are exercise echocardiography, also known as cardiopulmonary exercise testing, nailfold capillaroscopy, and molecular biomarkers [24]. Identification of the true predictive values for PAH can include systematic catheterization in future screening studies [24]. As a guideline, symptomatic patients with a high TRV with or without secondary echocardiographic signs of PAH or those who test positive on the DETECT algorithm or other pulmonary function test algorithms should undergo right heart catheterization [24].

Systemic sclerosis-associated interstitial lung disease (SSc-ILD)

ILD signifies a wide range of illnesses defined by improper gas exchange due to changes in the interstitial region of the lung’s architecture. ILD was predicted to have a yearly incidence of 30 per 100 000 people, with a female preponderance and a three-year average survival [35]. It occurs due to endogenous or exogenous causes, including environmental exposures, autoimmune diseases, infections, drugs, and radiation, with most cases being idiopathic. ILD can manifest as a dreaded complication in systemic sclerosis patients [13] as it does in 25%-30% of SSc patients with lung fibrosis [36].

**Figure 2:**
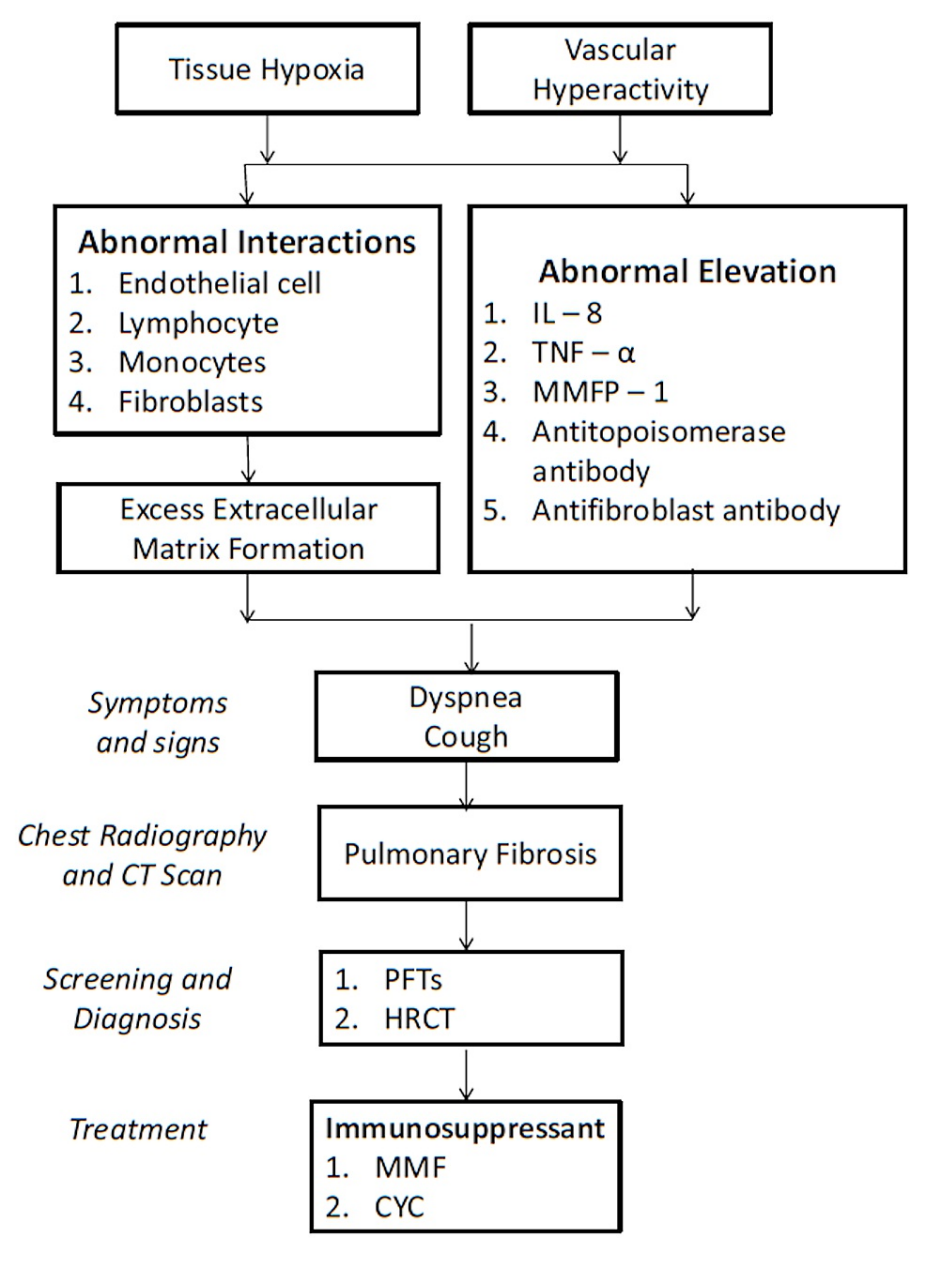
Summary of etiology, clinical features, and management of interstitial lung disease (ILD) in systemic sclerosis (SSc), SSc-ILD

Anti-Scl-70 antibodies, diffuse cutaneous SSc, African-American race, older age, and shorter disease duration have all been major risk factors for the development of SSc-ILD, according to observational studies [37-39]. Considering the pathophysiology behind SSc-ILD, involving tissue hypoxia and vascular hyperreactivity, there are abnormal interactions between endothelial cells, lymphocytes, monocytes, and fibroblasts resulting in excess of extracellular matrix formation by fibroblasts (Figure 2) [40]. Also, anti-fibroblast antibodies have been found to activate fibroblasts and stimulate extracellular matrix synthesis [41]. Pro-inflammatory cytokines interleukin-8 (IL-8), tumor necrosis factor-a (TNF-a), and macrophage inflammatory protein-1 were found to be elevated [42]. Patients with SSc-ILD have greater levels of anti-topoisomerase and anti-fibroblast antibodies, suggesting that B-cells may be implicated [43,44].

SSc has multiple manifestations clinically with dyspnea, cough, and a non-specific interstitial pneumonia pattern on computerized tomography (CT) scan as the most prevalent signs of systemic sclerosis-associated ILD, with only a small percentage of cases meeting the criteria for typical interstitial pneumonia [45]. SSc-ILD usually presents with pronounced pulmonary fibrosis in basilar portions of the lungs when seen on high-resolution CT (HRCT) or chest radiographs [46]. When evaluated, the radiographic extent of the disease and the likelihood of progression are two methods of many used for classifying SSc-ILD. Goh et al. created a categorization system to assess the severity of ILD in SSc in 2008 [41]. They divided patients into limited and extensive disease categories using a combination of HRCT and PFTs [47]. Patients with SSc-ILD can also be divided into groups based on disease progression [48]. Patients with a slow, gradual reduction in forced vital capacity (FVC) or stability and those with a fast progressive clinical picture will require lung transplantation or progress to eventual death regardless of therapy [48]. The disease's clinical presentation is varied and requires a thorough clinical analysis [48].

A comprehensive diagnosis becomes necessary for evaluating SSc patients based on the heterogeneous presentation of this disease [49]. Spirometry and single-breath carbon monoxide diffusion capacity (DLCO) are among the pulmonary function tests (PFTs) exclusively used in the diagnostic workup [49]. Minor alterations in lung function can be identified early, which helps determine the respiratory pattern before symptoms or chest radiography changes can arise [49]. The most prevalent alteration in PFTs is a lower FVC with normal or even increased volume that has been exhaled at the end of the first second of forced expiration (FEV1)/FVC ratio, indicating a restrictive ventilatory pattern [49]. Similarly, HRCT is a non-invasive method of diagnosing ILD since it can determine the nature and degree of pulmonary involvement [49].

On the evaluation of various therapeutic choices, immunosuppressants, particularly mycophenolate mofetil (MMF) or cyclophosphamide (CYC), have typically been the standard treatment [45]. When the two drugs were compared, MMF was better tolerated than CYC, as documented in the Scleroderma Lung Study II [50]. The researchers looked at 142 patients with SSc-ILD who had an FVC of 80% with ground glass opacities on HRCT [50]. The study concluded that MMF had a decreased incidence of leukopenia and thrombocytopenia, making it the first choice of treatment over CYC [50]. Based on the Scleroderma Lung Study II results, CYC is considered an alternative to MMF, comparable to earlier research [50]. Even though targeted biological and antifibrotic therapy can be used in addition to immunosuppressants [45], refractory cases still must be treated with autologous hematopoietic stem cell transplantation and lung transplantation [45].

Early screening is critical for SSc patients with ILD [51] since they tend to have comorbidities (Table 2) [52]. For the effective therapy of SSc-ILD patients, earlier detection and screening of pulmonary disease based on a recent reduction in lung function tests and the degree of lung involvement at high-resolution computed tomography become imperative [51]. The screening guidelines encompass a thorough examination of systems, a complete physical examination, a complete assessment of PFTs with lung volumes, and lung imaging with HRCT [51]. In a cohort study performed in the United States, on analysis of the incidence, prevalence, patient profile, immunosuppressive therapy (IST), and comorbid outcomes, it was found that out of 34,820 cases of SSc, 8252 cases of SSc-ILD were diagnosed. The study concluded that patients with SSc-ILD received more IST and had more significant comorbidities when compared to newly diagnosed SSc patients [52] (Table 2).

**Table 2:** Summary of included studies linking ILD and SSc. SSc, Systemic sclerosis; SSc-ILD, systemic sclerosis-associated interstitial lung disease; ILD, interstitial lung disease; IST, immunosuppressive therapy; NPV, negative predictive value; FVC, forced vital capacity; DLCO, single-breath carbon monoxide diffusion capacity; HRCT, high-resolution CT; PFT, pulmonary function test.

References	Design	Total cases of SSc	Cases of SSc-ILD	Study parameters	Diagnostic criteria	Conclusion
Li et al. (2021) [48]	Cohort study	34,820	8252	Incidence, prevalence, patient characteristics, immunosuppressive therapy (IST), and comorbid outcomes	In the SSc cohort, patients had one or more SSc diagnostic claims. Patients in the SSc-ILD cohort had an additional ILD claim.	In comparison to freshly diagnosed SSc, patients with SSc-ILD received more IST and had greater comorbidities.
Showalter et al. (2018) [52]	Cohort study	265	188	Sensitivity, specificity, and negative predictive value (NPV) of FVC and DLCO thresholds for SSc-ILD on HRCT	American College of Rheumatology 2013 SSc criteria with a chest HRCT scan and pulmonary function tests (PFT). FVC < 80% and DLCO < 62% for radiographic SSc-ILD.	High prevalence of radiographic ILD in SSc despite normal PFT values.
Sánchez-Cano et al. (2018) [53]	Descriptive multicentric cohort	1374	595	Clinical characteristics, differences according to the subtypes, mortality, and morbidity	The modified classification criteria of the American College of Rheumatology for SSc proposed by LeRoy and Medsger	ILD as a complication of SSc has significant morbidity and mortality in all three subgroups.

**Figure 3:**
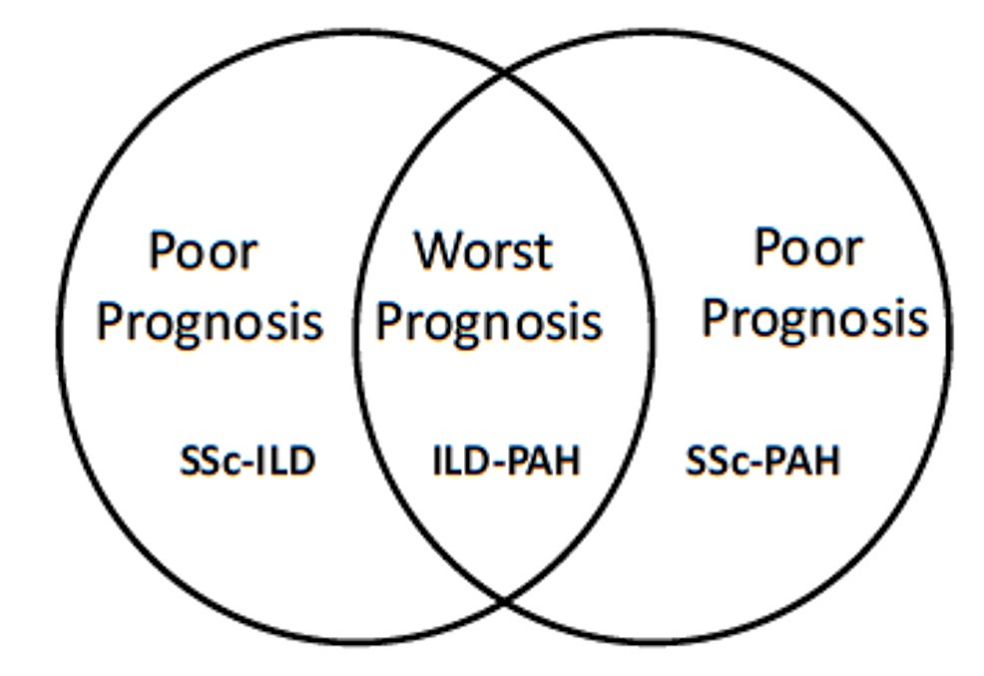
Prognosis of systemic sclerosis patients affected with ILD, PAH, and ILD associated with PAH (ILD-PAH). ILD, Interstitial lung disease; PAH, pulmonary arterial hypertension; SSc, systemic sclerosis.

With many studies describing the poor prognosis of SSc-ILD, there are many challenges we face in addressing the problem. There is a lack of consensus on ILD screening or disease progression monitoring despite the well-established link between SSc-ILD and mortality [54]. There is a lack of proven biomarkers for SSc-ILD and clinical recommendations to guide the methodology of investigations for diagnosis and identification of those at risk of progression [55]. ILD associated with pulmonary artery hypertension (ILD-PAH) patients had a worse five-year survival rate than SSc-PAH and SSc-ILD patients, with PAH being the only factor connected to an increased risk of mortality (Table 3) [56]. A substantial proportion of patients with SSc-related ILD also had PAH, making it critical to search for PAH in SSc-ILD patients (Table 3) [57]. Survival in SSc-PAH patients is poor, and SSc patients with ILD-related PAH have an even worse prognosis (Table 3) [58]. Patients with SSc-ILD or SSc-PAH have a poor prognosis, but the combination of ILD-PAH has the worst prognosis (Figure 3). Based on this analysis, a multidimensional approach to SSc-ILD should be devised, enabling early detection of ILD in SSc patients for timely intervention and a change in the disease trajectory.

**Table 3:** Summary of included studies linking ILD, PAH, and SSc. PAH, Pulmonary arterial hypertension; SSc, systemic sclerosis; SSc-ILD, systemic sclerosis-associated interstitial lung disease; ILD, interstitial lung disease; ILD-PAH, ILD associated with PAH; HRCT, high-resolution CT; sPAP, systolic pulmonary artery pressure; RHC, right heart catheterization; mPAP, mean pulmonary arterial pressure; PAH, pulmonary arterial hypertension; SSc-PAH, systemic sclerosis-associated pulmonary arterial hypertension.

References	Design	Total cases of SSc	Cases of SSc	Cases of SSc	Cases of SSc	Study parameters	Diagnostic criteria	Conclusion
			PAH	ILD	(ILD-PAH)			
Noviani et al. (2020) [55]	Cohort study	490	50	92	43	Mortality and survival	For ILD - HRCT - significant ILD - FVC <70% predicted PAH - echo (sPAP) ≥50 mmHg or RHC with mean PAP ≥25 mmHg. For mortality -multivariable regression analyses for survival - Cox proportional hazard model.	ILD-PH patients exhibited a lower five-year survival rate than SSc-PAH and SSc-ILD patients. Only PAH was linked to an increased risk of death on its own.
Young et al. (2019) [56]	Prospective observational cohort	93	50	24	29	Prevalence, characteristics, treatment, and outcomes	For PAH - right heart catheterization. For survival - Kaplan-Meier method was used.	A large proportion of patients with SSc-related ILD also had PH. Patients with SSc-related ILD should be investigated for PH as well.
Mathai et al. (2009) [57]	Cohort study	59	39	-	20	Determinants of survival	Right heart catheterization. Kaplan-Meier and Cox proportional hazards model	In SSc patients with PAH, survival is still dismal. SSc patients with ILD-related PAH have an especially poor prognosis. Early diagnosis and treatment may improve outcomes.

Limitations of the study

SSc has a complex network of etiologies with multiple underlying components. This article concentrates solely on SSC's respiratory problems for analysis and does not address other variables in the prognosis of this condition. Elevated pulmonary pressures in patients with SSc can be caused by various conditions, including PAH, parenchymal lung disease, and left heart disease. Not every one of them has been thoroughly investigated. Our article highlighted important studies for analysis but we were unable to address all of them.

## Conclusions

As addressed in this article, patients with SSc have a complex multisystem involvement with a wide range of clinical manifestations. The respiratory complications of SSc, notably ILD and PAH, can occur separately and are closely linked together, as evidenced by research. Despite recent medical advances, these complications are associated with a significant risk of mortality and a dismal prognosis. As a result, both respiratory complications should be screened in SSc patients. We notably discussed the difficulties that physicians confront when dealing with these illnesses. This article, we believe, can underline the critical necessity of early diagnosis of the disease and its sequela to improve the outcome. It can be accomplished through a strict screening routine, widespread availability of screening instruments, and general awareness of these illnesses, all of which will help the patient recover more quickly and have a better long-term prognosis. Finally, we believe that more significant research into the link between SSc and the respiratory system is needed to develop a more coordinated and direct approach to diagnosing and treating these disorders.
